# The Effects of Mixed Diets on Salivary Digestion

**Published:** 1899-02

**Authors:** 


### The Effects of Mixed Diets on Salivary Digestion.

The fourteenth ordinary meeting of this session of the Edinburgh Royal Society, says the *British Med. Journal,* was held on Monday, July 4th, Lord McLaren, Vice-President, in the chair. Dr. W. Aitchison Robertson read a paper on the "Effect of Mixed Diets on Salivary Digestion." The research was made with the object of determining whether the digestion of starch was affected favorably or unfavorably by its admixture with other articles of food. He found that porridge formed a fairly easily digested article of food, and that the greater the dilution of the porridge with water or milk the greater was its ease of digestion. In fact, well-made water-gruel was one of the most easily digested of foods. The reason why potatoes were so often badly borne by the stomach was probably due to the manner in which they were prepared. If, after being boiled, they were finely powdered, so that the saliva to gain ready access to the starch granules, they were easily and rapidly digested. On the other hand, when potatoes were sent to the table whole, the probability was that they would be imperfectly masticated and swallowed in fragment of greater or less size. In such a case the saliva could only act on the outer surfaces of the fragments, and the result was a very imperfect digestion. In respect of the digestion of bread, the experiments showed that it was much less acted upon by saliva when eaten alone than when taken along with some indifferent fluid as water. Newly baked bread was not as rapidly acted upon by saliva as stale bread, but the ultimate degree of starch conversion was greater in the former than in the latter. So far as the experiments showed, stale bread was not more easily digested than newly baked bread. The addition of milk to bread caused a remarkable enfeeblement of the salivary ferment, while broth exerted a slight restraining influence upon it. Alcohol, even in solution, retarded salivary digestion of starch, but the action was much less marked than in the case of the infusion of tea. Wines had a very marked inhibitory influence on the digestion of starch by saliva, and that was almost wholly due to their acidity. Even after three hours' digestion in the presence of sherry, port or claret, the starch had undergone hardly any conversion.—*Dietetic and Hygienic Gazette.*

